# Primary and metastatic tumor dormancy as a result of population heterogeneity

**DOI:** 10.1186/s13062-016-0139-0

**Published:** 2016-08-23

**Authors:** Irina Kareva

**Affiliations:** Simon A. Levin Mathematical, Computational and Modeling Sciences Center (SAL MCMSC), Arizona State University, Tempe, AZ USA

**Keywords:** Tumor dormancy, Quiescence, Population heterogeneity, Mathematical model

## Abstract

Existence of tumor dormancy, or cancer without disease, is supported both by autopsy studies that indicate presence of microscopic tumors in men and women who die of trauma (primary dormancy), and by long periods of latency between excision of primary tumors and disease recurrence (metastatic dormancy). Within dormant tumors, two general mechanisms underlying the dynamics are recognized, namely, the population existing at limited carrying capacity (tumor mass dormancy), and solitary cell dormancy, characterized by long periods of quiescence marked by cell cycle arrest. Here we focus on mechanisms that precede the avascular tumor reaching its carrying capacity, and propose that dynamics consistent with tumor dormancy and subsequent escape from it can be accounted for with simple models that take into account population heterogeneity. We evaluate parametrically heterogeneous Malthusian, logistic and Allee growth models and show that 1) time to escape from tumor dormancy is driven by the initial distribution of cell clones in the population and 2) escape from dormancy is accompanied by a large increase in variance, as well as the expected value of fitness-determining parameters. Based on our results, we propose that parametrically heterogeneous logistic model would be most likely to account for primary tumor dormancy, while distributed Allee model would be most appropriate for metastatic dormancy. We conclude with a discussion of dormancy as a stage within a larger context of cancer as a systemic disease.

**Reviewers:** This article was reviewed by Heiko Enderling and Marek Kimmel.

## Introduction

Long latency periods between treatment of primary tumors and appearance of metastatic disease suggest existence of tumor dormancy, which has been characterized as “cancer without disease” [1]. It appears that tumor dormancy can manifest itself not only in delayed metastatic recurrences [2–5] but also in primary tumors. Evidence for primary tumor dormancy is commonly found in autopsy studies, where men and women who died from trauma have been found to have a surprisingly high number of microscopic clinically unapparent tumors [6–9]. Furthermore, evidence from donor transplants has shown that microscopic tumors can be transmitted from donors who passed pre-transplantation screening as being free from cancer for over 10–15 years [10–14], further implicating the existence of cancer without disease.

A number of mechanisms have been proposed to account to explain the phenomenon of tumor dormancy. These include cell cycle arrest [15–17], tumor management by the immune system [18–20], age-dependent decreased relative fitness with respect to somatic cells [21], inability to secure blood supply [22–25], among others [15, 26, 27]. Unfortunately, there exist technical difficulties with investigating many of the hypotheses. Even if one is successful at isolating appropriate cell lines [22, 23, 28, 29], the typical experimental models involve mouse xenographs, which may or may not be applicable for these questions because of inter-species compatibilities. Development of a strong theoretical framework, including mathematical modeling, is thus necessary to further our understanding of this phenomenon.

Two main mechanisms for tumor dormancy are generally recognized: tumor mass dormancy and dormancy of solitary cancer cells, or quiescence [30–33]. Tumor mass dormancy is characterized by a balance of proliferation and apoptosis and can be viewed as the tumor having reached its pre-angiogenic carrying capacity [15]. Changes in the carrying capacity, whether due to variations in nutrient availability [34], or acquisition of the ability to recruit blood vessels and avoid immune surveillance [18], would allow for tumor escape. In contrast, solitary tumor dormancy, or quiescence, is characterized by lack of proliferation due to long periods of cell cycle arrest. While both mechanisms can lead to the phenomenon of latent tumor growth, Wells et al. argue based on their computer simulation study [35] that quiescence is a more likely mechanism to underlie metastatic dormancy.

In our previous work, we have looked at several examples of tumor growth being impacted by changes in dynamic carrying capacity [36]. Here, we will focus on studying a possible mechanism of escape from dormancy that stems not from external limitations but from intrinsic growth laws. Specifically, we propose that behavior consistent with escape from dormancy can come as a result of natural growth dynamics of heterogeneous populations. Therefore, we will focus on looking at the tumor dynamics before it reaches its carrying capacity as allowed by its microenvironment.

We will evaluate three different growth functions for heterogeneous populations. Specifically, we will look at parametrically heterogeneous populations that grow according to Malthusian, logistic and Allee growth laws. Several of these examples have been investigated in a different context by Karev [37]. We will demonstrate that a dynamics that is consistent with escape from tumor dormancy after a long period of latency can be generated due solely to heterogeneous nature of the populations described by these three models. In addition to changes in total population size, we will look at changes in expected values of intrinsic parameters, as well as variance and clone distributions, to demonstrate what alterations occur in the time period preceding escape from dormancy. We will conclude with a discussion of how this stage of tumor growth fits into a larger context of cancer as a systemic disease.

## Materials and methods

In order to investigate how dynamics consistent with tumor dormancy and subsequent escape from it can result solely from population heterogeneity, we will require the use of the parameter distribution technique, also known as the Reduction Theorem. It was well described in [37–40], and main results have been summarized in [41]. We will apply it to describing the dynamics of parametrically heterogeneous populations that grow according to Malthusian, logistic and Allee growth laws. Our goal here is to use methods developed by G. Karev in aforementioned publications to show how these results can be applied to understanding one of the possible mechanisms underlying tumor dormancy.

In our simulations, we will evaluate parameters necessary to reproduce dynamics consistent with escape from dormancy over approximately 10-year period (120 months). Since we are focusing on modeling tumor dynamics in the time preceding it reaching its carrying capacity, we are looking at the microscopic tumor reaching a size of approximately 1*mm*^3^, which is a limit imposed on non-angiogenic tumor size by oxygen and nutrient constraints [22]. We realize that there exist tissue and tumor-specific variations, and would like to emphasize that these estimates can be varied based on data. Our goal is to demonstrate that qualitative behavior of tumor dormancy and escape from it can be reproduced with these models.

### Parameters and distributions

#### Choice of initial distribution

According to the principle of maximum entropy (MaxEnt), if the mean value of the random variable is the only quantity that can be estimated from observations or other data, then the most likely distribution of the variable is exponential with the estimated mean [42]. Since no value of a biological characteristic, such as a growth rate, can be infinite, then we should choose the truncated exponential distribution in this interval as the initial distribution. Any other distribution can of course be used, if there exist data or theoretical considerations underlying its choice.

#### Parametrically heterogeneous Malthusian growth

Consider a population of cells *x*_*c*_(*t*), where each individual cell is characterized by an intrinsic growth rate *c*. A set of cells that are characterized by the same value of *c* is referred to as *c*-clone. The total size of the population is given by $N(t)={\displaystyle \sum_{\mathbb{A}}{x}_c}(t)$ if *c* takes on discrete values and $N(t)={\displaystyle {\int}_{\mathbb{A}}{x}_c(t)} dc$ if *c* is continuous. $\mathbb{A}$ denotes the range of possible values of parameter *c*.

The rate of change of *x*_*c*_(*t*) is given by

1 $$ {x}_c(t)\hbox{'}=c{x}_c(t). $$

Solution to this equation is *x*_*c*_(*t*) = *e*^*ct*^*x*_*c*_(0). Distribution of clones is by definition ${P}_c(t)=\frac{x_c(t)}{N(t)}$. From the definition of *N*(*t*) we can get that

2 $$ N(t)={\displaystyle {\int}_{\mathbb{A}}{x}_c}(t) dc={\displaystyle {\int}_{\mathbb{A}}{x}_c}(0){e}^{ct} dc=N(0){\displaystyle {\int}_{\mathbb{A}}{e}^{ct}}{P}_c(0) dc=N(0){M}_0\left[t\right], $$

where *M*_0_[*t*] = ∫_*A*_*e*^*ct*^*P*_*c*_(0)*dc* is by definition the moment generating function of the initial distribution of *c*-clones in the population.

It can be shown [37] that the expected value of parameter *c* is given by

3 $$ {E}^t\left[c\right]=\frac{d{M}_0\left[t\right]}{dt}/{M}_0\left[t\right] $$

and that variance of parameter *c* is given by

4 $$ Va{r}^t\left[c\right]=\frac{M_0\left[t\right]\hbox{'}\hbox{'}}{M_0\left[t\right]}-{\left(\frac{M_0\left[t\right]\hbox{'}}{M_0\left[t\right]}\right)}^2. $$

The equation for the rate of change of the total population size thus becomes

5 $$ \frac{dN}{dt}={E}^t\left[c\right]N(t), $$

with *E*^*t*^[*c*] defined above.

For truncated exponential distribution on the interval [0,1], the moment generating function is with parameter of exponential distribution *μ*, is given by

6 $$ {M}_0\left[t\right]=\left(\frac{\mu }{e^{\mu }-1}\right)\left(\frac{e^{\mu }-{e}^t}{\mu -t}\right), $$

the expected value is

7 $$ {E}^t\left[c\right]=\frac{e^t}{e^t-{e}^{\mu }}+\frac{1}{\mu -t} $$

and the variance is

8 $$ Va{r}^t\left[c\right]=\frac{1}{{\left(\mu -t\right)}^2}-\frac{e^{\mu +t}}{{\left({e}^{\mu }-{e}^t\right)}^2}. $$

As mentioned above, we are looking to reproduce the growth dynamics that commences with a period of very slow growth for a period of approximately 120–240 months (10–20 years), which then rapidly increases, reaching a size of ≈ 1*mm*^3^ as allowed by oxygen and nutrient limitations [22]. Here, we refer to the transitional phase, during which population size increases rapidly, as the *escape phase* of tumor growth.

**Fig. 1:**
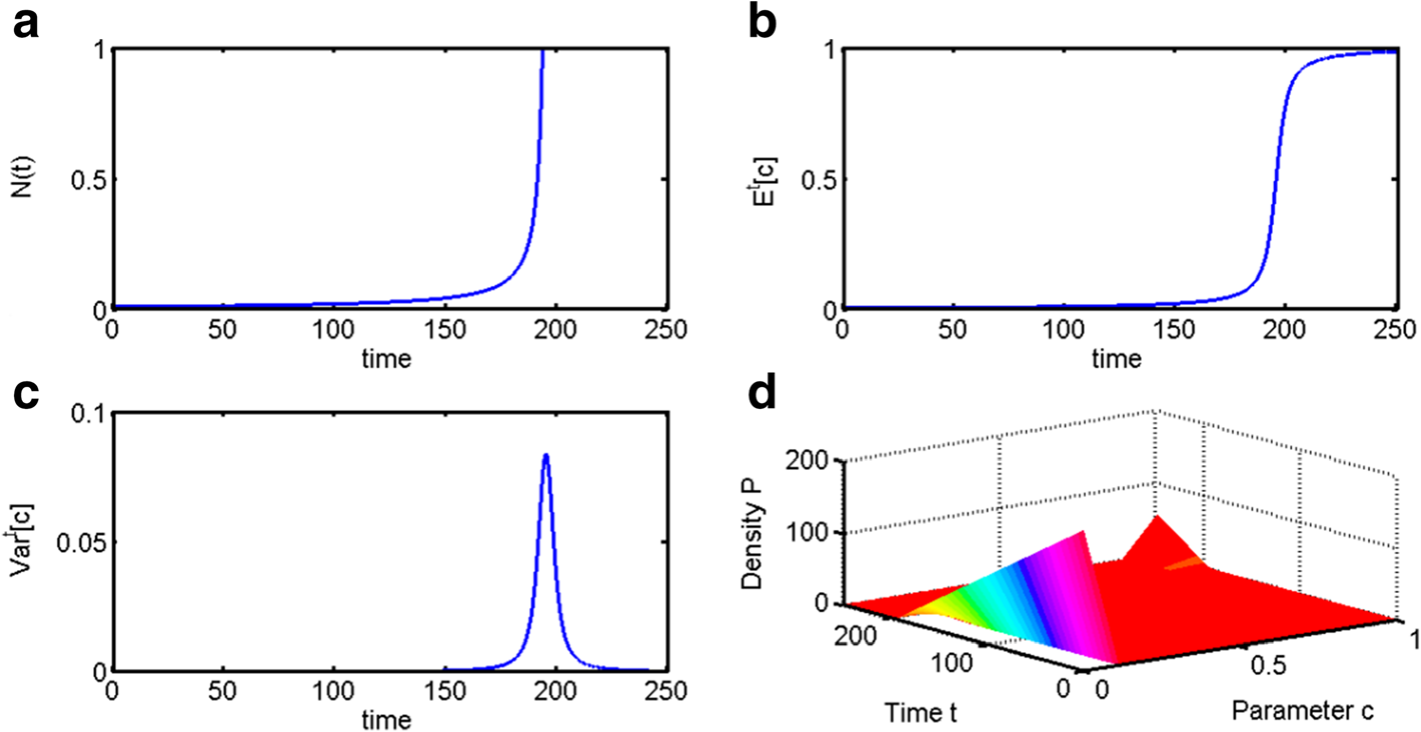
Parametrically heterogeneous Malthusian growth model with respect to growth rate parameter *c*. Initial distribution is taken to be truncated exponential, with distribution parameter *μ* = 195. Initial population size is *N*(0) = 0.001. **a** Population of cells N(t), which remains at a negligible size until *t* ≈ 180. **b** The expected value *E* ^*t*^[*c*] of Malthusian parameter *c* increases rapidly around the same time as the escape phase of tumor growth. **c** Variance *Var* ^*t*^[*c*] also increases dramatically during the escape phase but then decreases the clone with the largest allowed value of *c* becomes selected (in this case it is *E* ^*t*^[*c*] → 1, since the exponential distribution was taken to be truncated exponential on the interval *c* ∈ [0, 1]). **d** As the transition occurs, we can also see the distribution of cell clones change over time

As one can see in Fig. 1, even a simple parametrically heterogeneous Malthusian growth model can reproduce this effect. Until *t* ≈ 180, we can observe a period of growth, where the growth rate *E*^*t*^[*c*] and the population size *N*(*t*) increase very slowly. We will refer to this dynamics as *dormancy phase*. The dormancy phase is followed by a transitional *escape phase*, where population size increases very rapidly. In this example, where the parameter of truncated exponential distribution is *μ* = 195, the escape phase occurs at *t* ≈ 180 months = 15 years. During the escape phase, we can observe a characteristic bell-shaped graph of *Var*^*t*^[*c*], where variance increases together with population size *N*(*t*) and the expected value *E*^*t*^[*c*], and then decreases again as the clones with the highest fitness become selected. In this model, clones with the highest fitness are the ones with the largest possible value of parameter *c*, which is determined by the maximal value of growth rate parameter *c* in on the interval, on which the initial distribution was defined.

**Fig. 2:**
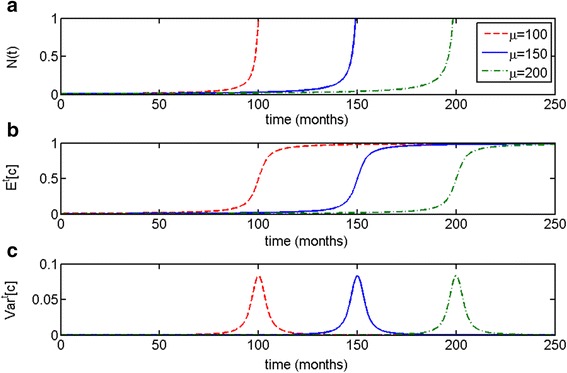
Parametrically heterogeneous Malthusian growth model with respect to growth rate parameter *c*. Initial distribution is taken to be truncated exponential, with *μ* = 100 (*dotted, red*) 150 (*solid, blue*) and 200 (*dash-dot, green*). Initial population size is *N*(0) = 0.001. As one can see, the patterns of behavior for (**a**) population size *N*(*t*), (**b**) expected value *E* ^*t*^[*c*] and (**c**) variance *Var* ^*t*^[*c*] remain qualitatively the same. Time to the escape phase is determined by the initial composition of the population, determined by *μ*

As one can see in Fig. 2, it is the initial composition of the population, determined by the value of the parameter of the initial distribution *μ*, which correlates with time to the escape phase (larger value of *μ* corresponds to longer time to escape phase). This pattern of correspondence of population variance and the expected value of the Malthusian growth parameter to time to the escape phase, remains the same regardless of the value of *μ*.

Even though parametrically heterogeneous Malthusian model already allows reproducing behavior that is qualitatively similar to tumor dormancy and subsequent escape from it, the primary issue with it of course lies in the fact that it allows unrestrained growth. However large the growth rate parameter may be, there always exist limitations on final population size, whether they be space or nutrient-related, among others. A microtumor cannot grow past a size allowed by the organ and blood supply limitations, rendering logistic growth model more applicable.

#### Parametrically heterogeneous logistic growth

Now let us consider a parametrically heterogeneous case of the logistic growth model, which addresses the issue of uncontrollable growth in finite time of the Malthusian model. Similarly to the previous case, we are looking at a population of cell clones *x*_*c*_(*t*), where each individual cell is characterized by an intrinsic growth rate *c*. The full equation is given by

9 $$ {x}_c(t)\hbox{'}=c{x}_c(t)\left(1-\frac{N(t)}{K}\right), $$

where *N*(*t*) is the total population size and *K* is the carrying capacity.

Let us introduce an auxiliary variable *q*(*t*), such that

10 $$ \frac{dq(t)}{dt}=1-\frac{N(t)}{K}. $$

Then

11 $$ \frac{d{x}_c(t)}{x_c(t)}=cq(t)\hbox{'} $$

and therefore

12 $$ {x}_c(t)={x}_c(0){e}^{cq(t)}. $$

We can calculate total population size to be

13 $$ N(t)={\displaystyle {\int}_{\mathbb{A}}{x}_c}(t) dc={\displaystyle {\int}_{\mathbb{A}}{x}_c}(0){e}^{cq(t)} dc=N(0){\displaystyle {\int}_{\mathbb{A}}{P}_c}(0){e}^{cq(t)} dc=N(0){M}_0\left[q(t)\right], $$

which follows from the definition of moment generating function *M*_0_[*q*(*t*)] for the initial distribution *P*_*c*_(0). Since in this case, the moment generating function of the truncated exponential distribution on the interval [0,1] is ${M}_0\left[q(t)\right]=\left(\frac{\mu }{e^{\mu }-1}\right)\left(\frac{e^{\mu }-{e}^{q(t)}}{\mu -q(t)}\right)$, the final equation for *q*(*t*) ' becomes

14 $$ \frac{dq(t)}{dt}=1-\frac{N(0)}{K}{M}_0\left[q(t)\right]=1-\left(\frac{N(0)}{K}\right)\left(\frac{\mu }{e^{\mu }-1}\right)\left(\frac{e^{\mu }-{e}^{q(t)}}{\mu -q(t)}\right), $$

which fully closes the system.

The distribution of clones over time is given by

15 $$ {P}_c(t)=\frac{x_c(t)}{N(t)}=\frac{x_c(0){e}^{cq(t)}}{N(0){M}_0\left[q(t)\right]}. $$

**Fig. 3:**
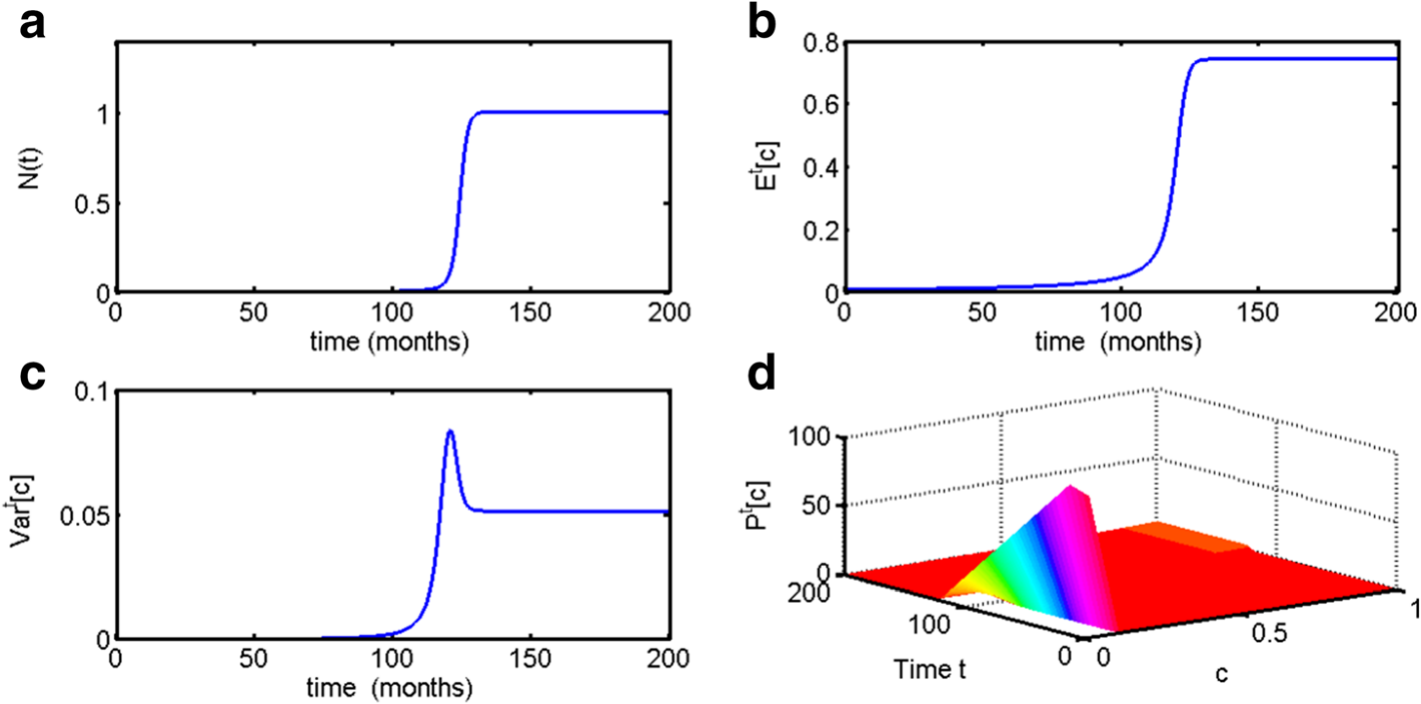
Parametrically heterogeneous logistic growth model with respect to growth rate parameter *c*. Parameter of truncated exponential distribution on the interval *c* ∈ [0, 1] is taken to be *μ* = 120, initial population size is *N*(0) = 0.001, *K* = 1. As can be seen, (**a**) in this case population escapes dormancy at *t* ≈ 120 months = 10 years. **b** The escape phase is accompanied by rapid increase in the expected value of *c*, as well as (**c**) a rapid increase in variance, which remains at a non-zero value even when the population is at a steady state. **d** The distribution of clones over time also changes away from the initial composition, albeit less dramatically than in the parametrically heterogeneous Malthusian model

As one can see in Fig. 3, the qualitative behavior in the time preceding escape from dormancy is similar to that of the parametrically heterogeneous Malthusian model (Fig. 3a) with the distinction of a limiting size being reached after the escape phase. For *μ* = 120 it occurs *t* ≈ 120 months = 10 years. The pattern of behavior of the expected value *E*^*t*^[*c*] is qualitatively similar to that of the Malthusian model, although in this case it does not reach the maximum value, allowed by the interval of the truncated exponential distribution, and remains below 1 (Fig. 3b). The dynamics of the variance *Var*^*t*^[*c*] is however qualitatively different from the previous case. It also increases dramatically in the moments preceding escape from dormancy. However, it remains consistently at a non-zero value even after the population has reached its carrying capacity. Unlike the Malthusian model, in the parametrically heterogeneous logistic growth model, the population maintains heterogeneity at equilibrium and does not select for a single clone.

**Fig. 4:**
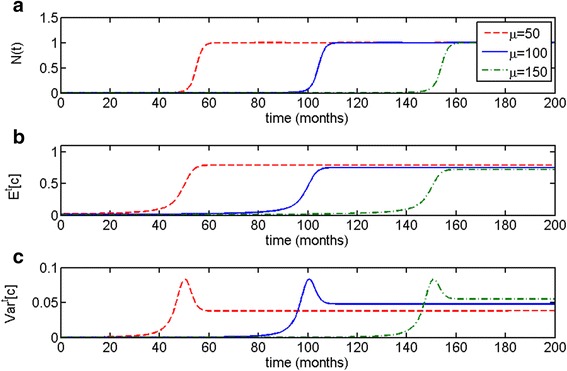
Parametrically heterogeneous logistic growth model with respect to growth rate parameter *c*. Parameter of truncated exponential distribution on the interval *c* ∈ [0, 1] is taken to be *μ* = 120, initial population size is *N*(0) = 0.001, *K* = 1. **a** Variations in the value of parameter *μ* determine time to escape from dormancy, which is (**b**) accompanied by increase in the expected value *E* ^*t*^[*c*] and (**c**) increase and stabilization in variance *Var* ^*t*^[*c*]. Noticeably, the higher the value of *μ* and the later the onset of the escape phase, the higher the variance at the steady state

Similarly to the distributed Malthusian model, time to escape from dormancy is determined primarily by the initial distribution of the population (see Fig. 4). Noticeably, the higher the value of *μ* and the later the onset of the escape phase, the higher the variance at the steady state, as can be proven through formulas (7) and (8).

**Fig. 5:**
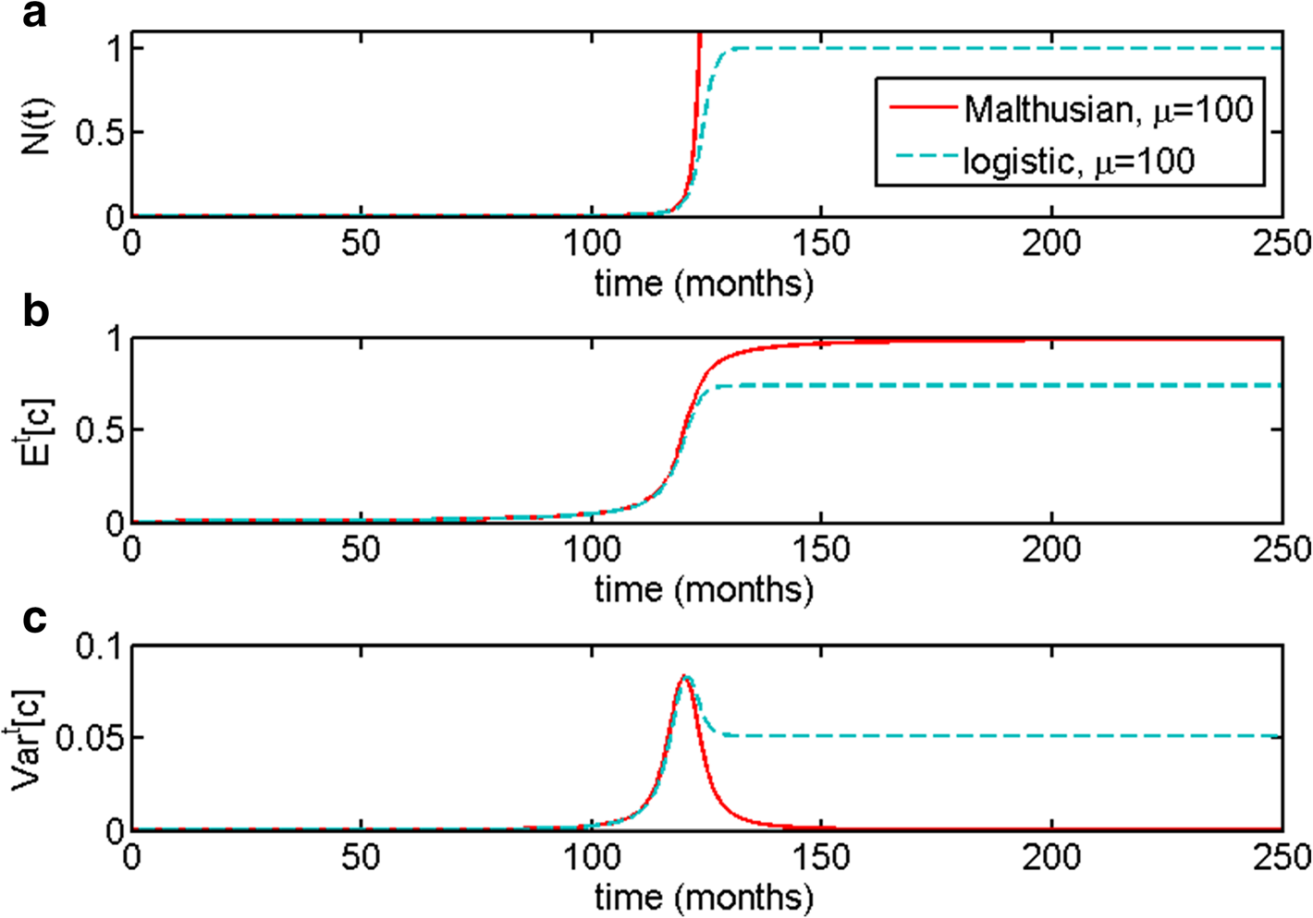
Comparison of distributed Malthusian and logistic growth models, with *N*(0) = 0.001, and with truncated exponential initial distribution on the interval *c* ∈ [0; 1] with parameter of the distribution being *μ* = 100. For the logistic model, *K* = 1. As one can see, dynamics up to the escape phase are identical but at the steady state, the logistic population maintains heterogeneity and consequently lower final *E* ^*t*^[*c*]

Comparison of the two cases, namely, the parametrically heterogeneous Malthusian and logistic models, can be found in Fig. 5. As one can see, for identical initial distributions and initial conditions, behavior in the time preceding the escape phase is very similar. During the escape phase, we can observe increase in the expected value of the parameter *c* and rise in variance in both models. However, in the case of a parametrically heterogeneous logistic population, *Var*^*t*^[*c*] > 0 is maintained even at the steady state, which is more consistent with our understanding of tumor biology.

As a point of observation, one can see that as *t* → ∞, *q*(*t*) → *q* *, where *q* * is some constant. Therefore, as *t* → ∞, the population *N*(*t*) grows like a Malthusian model at a finite moment in time, which allows maintaining population heterogeneity. One could conceivably interpret *q*(*t*) as the “internal time” of the population, since with respect to *q*(*t*), growth of total population becomes Malthusian, with each clone growing irrespective of the others [43]. One can interpret this observation as a tumor growing according to some “internal clock” that may be different from that of the surrounding tissues.

#### Parametrically heterogeneous Allee growth

Finally, let us consider a model of Allee-type growth. The general form is

16 $$ {x}_i\hbox{'}=c{x}_i\left(l-N\right)\left(N-m\right),i=c,l,m $$

where *N*(*t*) is the population size, *l* is the carrying capacity of the population, *m* is the meta-stable point that divides areas of attraction of the two equilibria 0 and *m*. Here, we will consider Allee-type models, heterogeneous with respect to each of the three parameters. We will first do the transformation for all three models, and then compare their behavior.

#### Distribution of growth parameter c

Similarly to the previous cases, consider a population of clones *x*_*c*_(*t*) that are characterized by an intrinsic heritable value of parameter *c*. Introduce an auxiliary “keystone” variable *q*_1_(*t*) such that

17 $$ {q}_1(t)\hbox{'}=\left(l-N(t)\right)\left(N(t)-m\right). $$

Then

18 $$ {x}_c(t)={x}_c(0){e}^{c{q}_1(t)}, $$

19 $$ N(t)=N(0){M}_0\left[{q}_1(t)\right], $$

20 $$ {P}_t\left[c\right]={P}_0\left[c\right]\frac{e^{c{q}_1(t)}}{M_0\left[{q}_1(t)\right]}. $$

The expected value *E*^*t*^[*c*] and variance *Var*^*t*^[*c*] of the population are defined through the moment generating function of the initial distribution according to formulas (3) and (4), respectively.

**Fig. 6:**
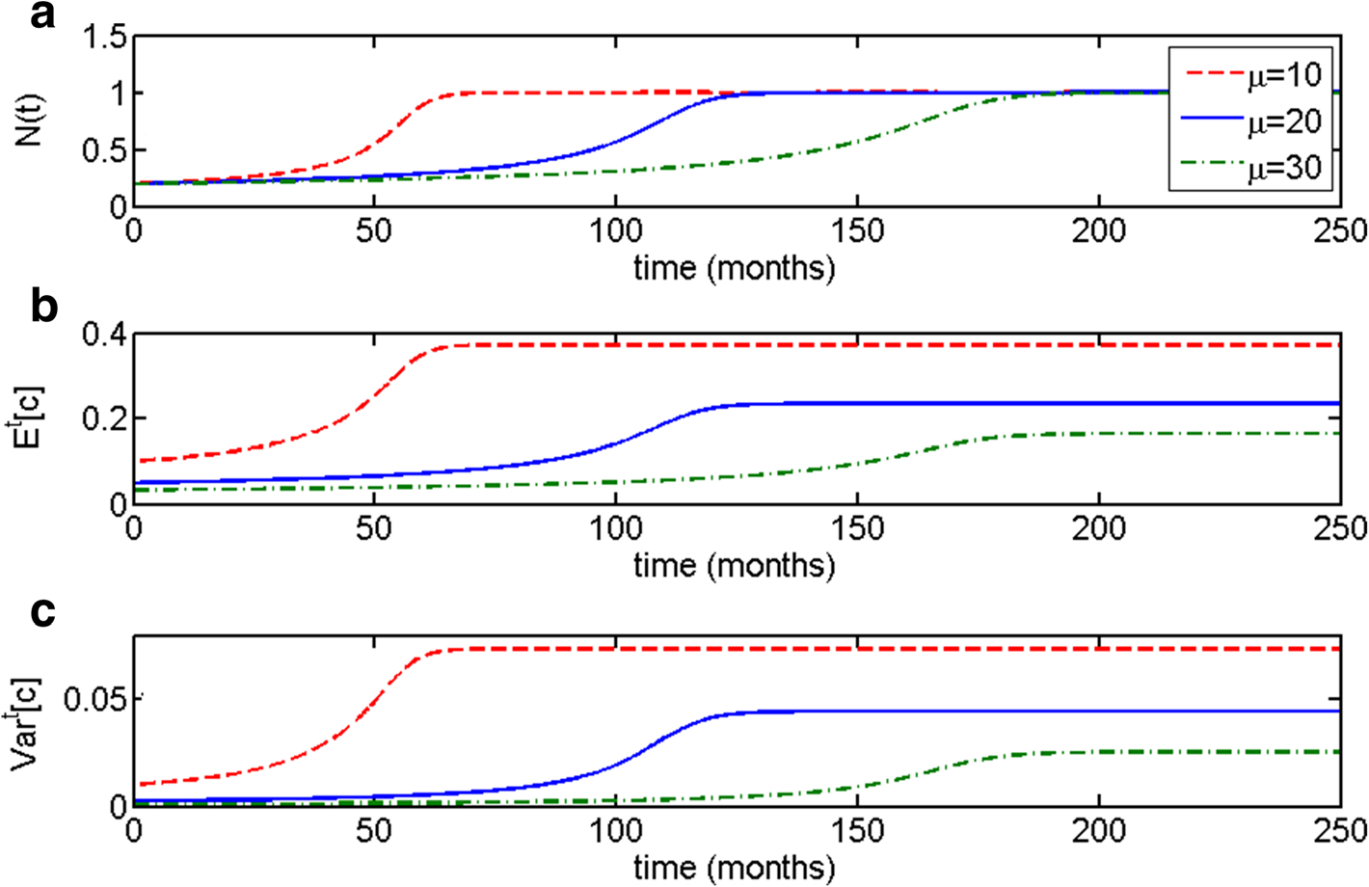
Allee growth model with distributed growth parameter *c* (c-distributed Allee model). The initial distribution is truncated exponential on the interval *c* ∈ [0, 1]. Other parameters are *m* = 0.1, *l* = 1; the initial population size is *N*(0) = 0.2. **a** Time to escape is determined by the initial population composition, given by parameter *μ*. **b** Expected value of *c* increases simultaneously with population size, (**c**) as does variance *Var* ^*t*^[*c*]. Noticeably, later escape in this example correlates with lower variance at the steady state

As one can see in Fig. 6, escape from dormancy in these types of models is more gradual compared to Malthusian and logistic cases. The escape phase occurs later with higher values of *μ*, and population growth curves increase more or less gradually depending on the value of *μ* (Fig. 6a). Similarly to previous cases, the expected value of *c* starts to increase with population size (Fig. 6b), as does the variance (Fig. 6c). As in the case of the distributed logistic model, there is a correlation between time to escape and variance: the higher the value of *μ*, the later the onset of the escape phase (such as for *μ* = 30) and the lower the predicted variance *Var*^*t*^[*c*] at the steady state, as can be shown through formulas (7) and (8).

This kind of gradual escape might account for the dynamics of slower growing tumors, such as some prostate cancers [44]. This hypothesis warrants further investigation.

#### Distribution of carrying capacity l

Now consider a population of cells *x*_*l*_ that differs in the value of the carrying capacity *l*. Rewrite Eq. (16) as

21 $$ \frac{d{x}_l(t)}{dt}=c{x}_l(t)\left(l-N(t)\right)\left(N(t)-m\right)=c{x}_l(t)\left(l\left(N(t)-m\right)-N(t)\left(N(t)-m\right)\right)dt $$

Now define the following keystone variables:

22 $$ \begin{array}{l}\frac{dp}{dt}=c\left(N(t)-m\right),\\ {}\frac{dq}{dt}=cN(t)\left(N(t)-m\right).\end{array} $$

Then $\frac{d{x}_l(t)}{dt}={x}_l(t)\left(l\frac{dp}{dt}-\frac{dq}{dt}\right)$ and consequently

23 $$ {x}_l(t)={x}_l(0){e}^{lp(t)-q(t)}. $$

Total population size then is given by

24 $$ N(t)={\displaystyle {\int}_L{x}_l}(t) dl={\displaystyle {\int}_L{x}_l}(0){e}^{lp(t)}{e}^{-q(t)} dl={N}_0{e}^{-q(t)}{\displaystyle {\int}_L{P}_0}(l){e}^{lp(t)} dl={N}_0{e}^{-q(t)}{M}_0\left[p(t)\right]. $$

The distribution of clones is

25 $$ {P}_l\left[t\right]=\frac{x_l(t)}{N(t)}=\frac{x_l(0){e}^{lp(t)-q(t)}}{N_0{e}^{-q(t)}{M}_0\left[p(t)\right]}={P}_l\left[0\right]\frac{e^{lp(t)}}{M_0\left[p(t)\right]}. $$

The expected value of *l* is ${E}^t\left[l\right]=\frac{M_0\left[p(t)\right]\hbox{'}}{M_0\left[p(t)\right]}.$

The characteristics of the full system can thus be given by Eq. (17), with *N*(*t*) defined above by Eq. (18).

**Fig. 7:**
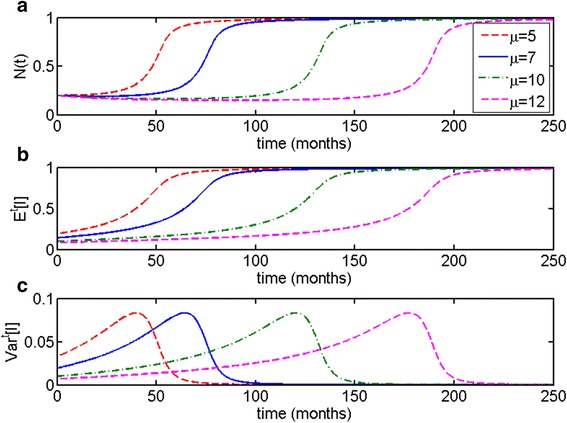
Allee growth model with distributed carrying capacity *l* (*l*-distributed Allee model). The initial distribution is truncated exponential on the interval *l* ∈ [0, 1]. Other parameters are *m* = 0.1, *c* = 1; the initial population size is *N*(0) = 0.2. **a** Time to escape is determined by initial population composition, given by the parameter *μ*. **b** Expected value *E* ^*t*^[*l*] increases simultaneously with population size, (**c**) as does variance *Var* ^*t*^[*l*]. Noticeably, variance increases much more gradually compared to the previous cases, and decreases rapidly after the population reaches a steady state

In the *l*-distributed Allee model, increase in population size during the escape phase is steeper (Fig. 7), similarly to the logistic and Malthusian cases. Time to escape is once again determined by initial population composition. One can see that for larger *μ*, population size decreases slightly before the escape phase (Fig. 7a). In this model, variance increases during the escape phase concurrently with both population size and *E*^*t*^[*l*], but unlike most previous cases, rapidly decreases to zero when the population has reached a steady state. Since the *l*-distributed Allee model does not maintain heterogeneity at a steady state, it is less likely to be an appropriate model for our question.

#### Distribution of parameter m

Finally, consider the case, where each clone is characterized by an individual value of parameter *m*. Here, the keystone equations are

26 $$ \begin{array}{l}q(t){\textstyle \hbox{'}}=N(t)c\left(l-N(t)\right),\\ {}p{\textstyle \hbox{'}}(t)=-c\left(l-N(t)\right).\end{array} $$

Consequently, $\frac{x_m(t)\hbox{'}}{x_m(t)}=q(t)\hbox{'}+mp(t)\hbox{'}$, and *x*_*m*_(*t*) = *x*_*m*_(0)*e*^*q*(*t*) + *mp*(*t*)^.

The total population size is given by

27 $$ \begin{array}{l}N(t)={\displaystyle {\int}_{\mathbb{M}}{x}_m}(0){e}^{q(t)}{e}^{mp(t)} dm={\displaystyle {\int}_{\mathbb{M}}{P}_m}(0)N(0){e}^{q(t)}{e}^{mp(t)} dm=\\ {}\kern2.25em =\kern0.5em N(0){e}^{q(t)}{\displaystyle {\int}_{\mathbb{M}}{P}_m}(0){e}^{mp(t)} dm=N(0){e}^{q(t)}{M}_0\left[p(t)\right].\end{array} $$

**Fig. 8:**
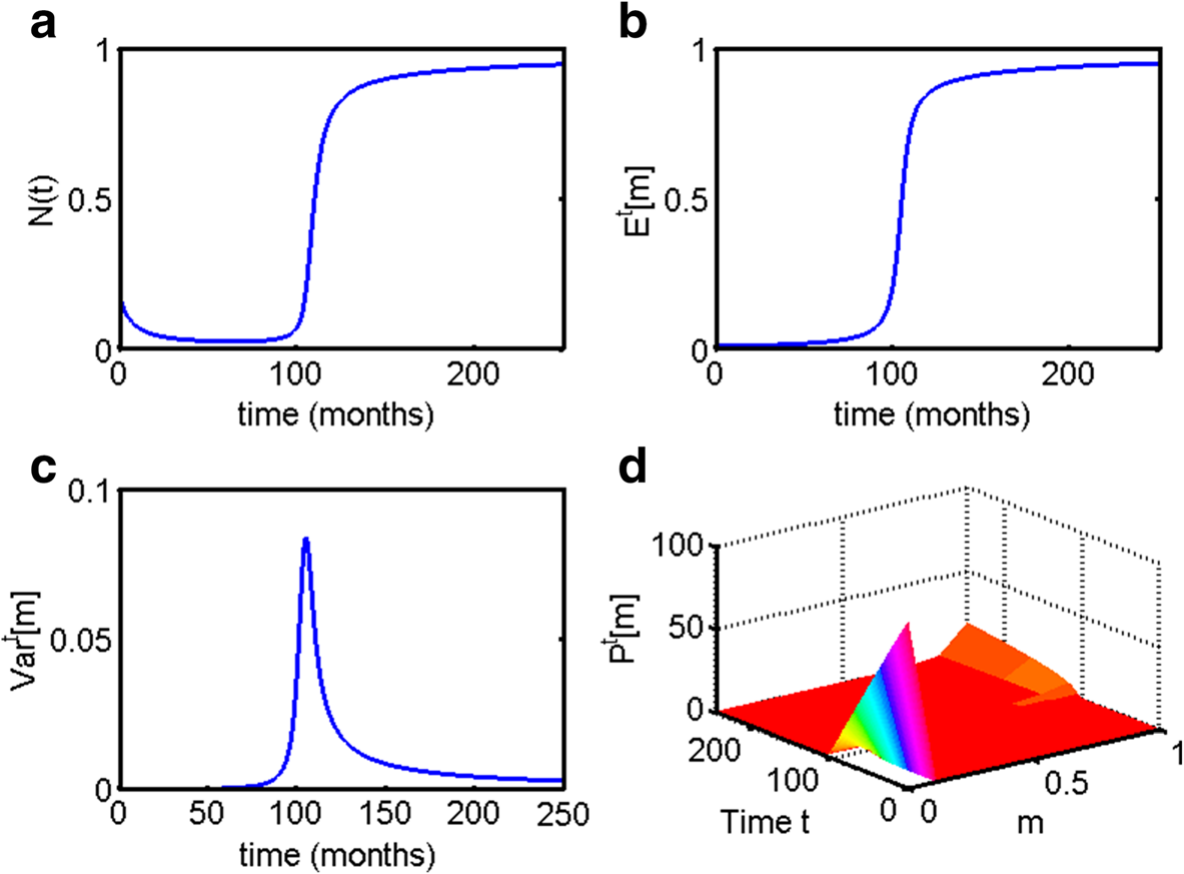
Allee growth model with distributed parameter *m* (*m*-distributed Allee model). The initial distribution is truncated exponential on the interval *m* ∈ [0, 1], with parameter of the distribution being *μ* = 100. Other parameters are *c* = 1, *l* = 1; the initial population size is *N*(0) = 0.2. **a** Total population size *N*(*t*) decreases in the months and years preceding the escape phase. **b** The expected value of *m* increases during the escape phase, (**c**) as does variance of *m*. However, *Var* ^*t*^[*m*] decreases dramatically at an equilibrium state. **d** Distribution of clones also changes noticeably over time, selecting for clones with higher values of *m*

**Fig. 9:**
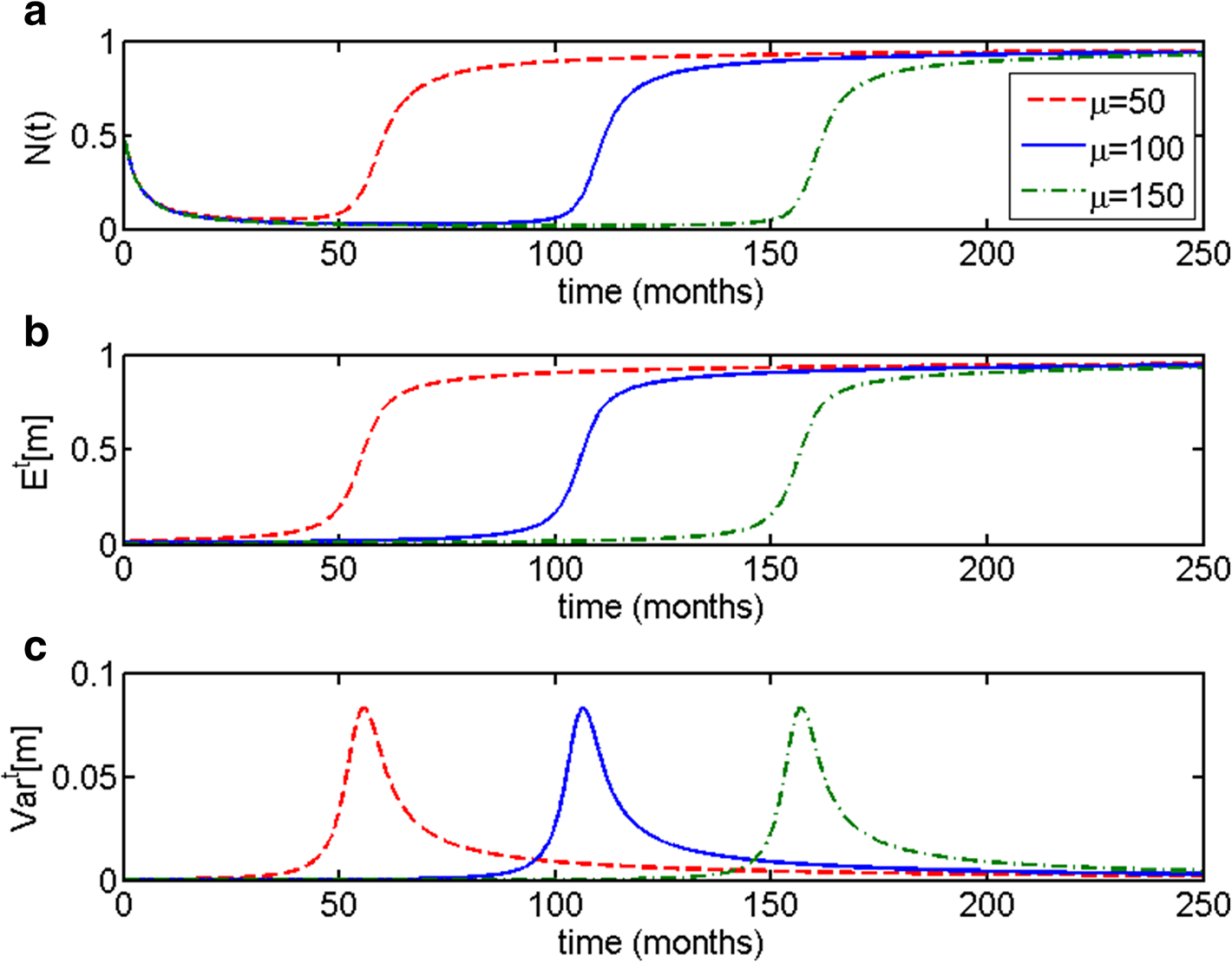
Allee growth model with distributed parameter *m* (*m*-distributed Allee model). The initial distribution is truncated exponential on the interval *m* ∈ [0, 1]. Other parameters are *c* = 1, *l* = 1; the initial population size is *N*(0) = 0.2. **a** Time to escape is determined by the initial population composition, given by the parameter *μ*. In the months and even yeas preceding escape from dormancy we can observe a sustained decrease in population size. **b** Expected value of *m* increases simultaneously with population size, (**c**) as does variance *Var* ^*t*^[*m*]. Noticeably, variance increases more gradually compared to the previous cases, and decreases slowly after the population reaches a steady state, compared to the l-distributed Allee model

As one can see in Fig. 8, populations that grow according to the *m*-distributed Allee model exhibit a unique behavior: the population size can decrease dramatically in the months and even years preceding the escape phase (Fig. 8a). Escape phase is once again accompanied by increase in the expected value of *m* (Fig. 8b), increase in variance (Fig. 8c) and change in clone distribution (Fig. 8d). Noticeably, *Var*^*t*^[*m*] decreases after the population reaches a steady state, with the equilibrated population becoming more homogeneous over time. Furthermore, similarly to the previous cases, the pattern of behavior remains consistent for various initial population compositions, as can be seen in Fig. 9. In this case, even though population heterogeneity decreases after the population has reached its carrying capacity, the decline in *Var*^*t*^[*m*] occurs so slowly that it may conceivably represent the dynamics of metastatic dormancy, where eventual selection for fewer clones and thus a gradual decrease in heterogeneity is expected to occur [45].

#### Comparison of the three distributed Allee models

**Fig. 10:**
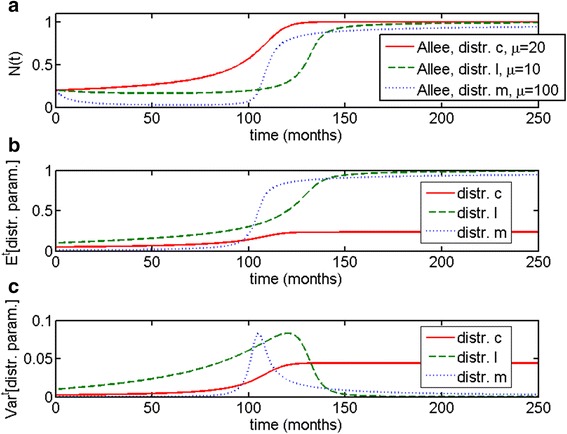
Comparison of the three parametrically heterogeneous Allee growth models. The initial distribution is truncated exponential on the interval [0, 1] for all three models. Parameters are *c* = 1, *l* = 1, *m*=.1 for each respective model; the initial population size is *N*(0) = 0.2. **a** The total population size *N*(*t*) for all three models, with *c*-distributed Allee model increasing most gradually, *l*-distributed Allee model remaining at a steady size before increasing, and *m*-distributed Allee model predicting a decrease in population size to near-zero until the escape phase. **b** Expected value of each of the parameters for their respective models and (**c**) variance of each of the parameters for their respective models. In every case, escape phase is accompanied by increase in both the expected value and variance. The *c*-distributed model maintains heterogeneity at a steady state; *l*-distributed model rapidly becomes more homogeneous at the steady state; *m*-distributed model slowly gradually loses heterogeneity over time after the escape phase

Now, let us compare all three parametrically heterogeneous Allee growth models. All the examples in Fig. 10 were chosen to describe escape from dormancy at approximately *t* = 100 to *t* = 120 months, or 8.3–10 years. All three models provide dynamical behaviors that are consistent with escape from dormancy.

Escape phase predicted by *c*-distributed Allee model occurs in the most gradual way out of all of the examples, and is accompanied by slight increase in the expected value of *c* and a steady *Var*^*t*^[*c*] at equilibrium. A population that grows according to *l*-distributed Allee model exhibits sharper increase in population size during the escape phase; its final population composition is the most homogeneous, with selection towards the largest value of *l*.

Finally, a population that grows according to the *m*-distributed Allee model exhibits a more unusual dynamics, with population size *N*(*t*) dropping to near-zero and remaining dormant for many months and years before eventually rapidly increasing in size. Like in every case described, the escape phase in all of the populations is accompanied by increase in both the expected value of the distributed parameter, and in the variance. In this case, the population becomes less heterogeneous over time but does so much more gradually, compared to population with distributed carrying capacity *l*.

## Discussion

We propose that long latency periods between appearance of cancer cells and disease manifestation, whether for a primary tumor, or for a metastatic recurrence, could be accounted for by tumor heterogeneity. We investigate this hypothesis using several parametrically heterogeneous mathematical models, including parametrically heterogeneous Malthusian, logistic and Allee-type growth models. Heterogeneity can be a result of either heritable characteristics, such as proliferation and apoptosis rates; mutually influential epigenetic variability, or epigenetic landscapes [46]; degree of adaptability to the environment [21]; or even genetic mosaicism [47]. Within the context of the models, such intrinsic variations were accounted for with parameters of initial distribution of cell clones in the population; these parameters may represent degree of epigenetic variability, sensitivity to hormones, or any other fitness-affecting characteristic of the initial tumor population, depending on tumor type.

In our simulations, we looked at populations of cells that undergo rapid growth after a prolonged period of latency, the dynamics that we refer to here as the escape phase. This escape from dormancy occurs after a simulated period of 5–20 years. We were looking for the simulated tumor to reach a volume of 1*mm*^3^ as allowed by oxygen and nutrient limitations [22], and are particularly interested in the dynamics that precedes the simulated tumor reaching its carrying capacity.

### Parametrically heterogeneous growth models: Malthusian, logistic and Allee

In our investigation, we have applied the Reduction theorem, which was developed by G. Karev [37–39], to three population growth models, namely, Malthusian, logistic and Allee. The Reduction theorem, or parameter distribution technique, allows subdividing the population into subpopulations of cells that are characterized by a particular value of some parameter. These subpopulations are referred to as clones. The distribution of clones changes over time, and this dynamics, as well as changes in the corresponding statistical characteristics, such as expected value of the parameter or its variance in the population, can be monitored using the moment generating function of the initial distribution. Investigations of parametrically heterogeneous Malthusian and logistic models were done in [37]; some of the results are re-interpreted here in the context of tumor growth.

Here, in Malthusian and logistic models, we distributed proliferation rate parameter *c*. In the Allee growth model, we investigated three cases: populations were assumed to be heterogeneous with respect either to proliferation parameter *c*, or carrying capacity parameter *l*, or parameter *m*, which divides the domain of attraction of the two stable equilibria of the initial model. In these examples, the initial distribution was taken to be truncated exponential, on the interval [0; 1], with distribution parameter *μ*.

The results can be summarized as follows. In all of the cases, the escape phase was accompanied by dramatic increase in both the variance and the expected value of the parameter under investigation. Time to the onset of the escape phase was determined by the initial composition of the population, as given by the value of parameter *μ*. The values of *μ* for different models varied from 5 to 200.

In the case of the Malthusian parametrically heterogeneous model, the population variance spiked during the escape phase but then declined dramatically, resulting in an uncontrollably growing population with the largest permissible value of parameter *c* (Figs. 1 and 2). In the logistic parametrically heterogeneous model, the population maintained a degree of heterogeneity at the equilibrium state, once it has reached its carrying capacity (Figs. 3 and 4), which is more likely in application to tumor growth, as tumors are known to be heterogeneous [45, 46, 48]. Distributed Malthusian and logistic models are compared in Fig. 5.

Distributing the logistic equation required introducing an auxiliary “keystone” variable *q*(*t*) (in parallel with keystone species in ecology, which have disproportionately large effect on the population relative to their abundance). This variable can be interpreted as the *“internal time*” of the population, since with respect to *q*(*t*), growth of total population becomes Malthusian, with each clone growing irrespective of the others [49]. Thus one can hypothesize that in this case, the population of tumor clones may be growing according to its own internal clock.

The Allee growth model with distributed proliferation parameter *c* (the *c*-distributed Allee model) also maintains population heterogeneity at an equilibrium state (Fig. 6). Moreover, in this case, the escape phase occurs very gradually compared to the other models. The Allee growth model with distributed carrying capacity parameter *l* (the *l*-distributed Allee model) does not maintain heterogeneity at the equilibrium state (Fig. 7); the escape phase occurs less gradually compared to the *c*-distributed Allee model but more so than any of the other models. The Allee growth model with distributed parameter *m* (the *m*-distributed Allee model) demonstrates the most unusual of the behaviors. In this case, one can observe decrease in population size to near-zero zero for a prolonged period of time before the escape phase (Figs. 7 and 8). Population heterogeneity decreases after the escape phase but not as rapidly as Malthusian or l-distributed Allee models. The behavior of this model is most consistent from a theoretical point of view with metastatic dormancy, as will be discussed further. A comparison of all three distributed Allee models is given in Fig. 10.

### Primary dormancy, metastatic dormancy, both, or neither?

While it is more likely that mechanisms governing metastatic dormancy differ from those underlying primary tumor dormancy, it is possible that a subset of metastatic dormant tumors, which have disseminated very early, might be growing according to growth laws proposed here.

A number of experimental models have suggested that metastatic spread can occur at very early stages of primary tumor development [50]. Newman and Cisneros [51] have investigated the question of whether, from a theoretical point of view, dissemination using few very specialized cells (“special forces”) or seeding a large number of non-specialized cells (“infantry”) would be more effective for metastatic colonization. Their analysis has shown that in fact both strategies are equally likely (or unlikely) to seed disseminated tumors. Therefore, it is possible that a tumor that has disseminated early [48, 50, 52, 53] might be growing according to the same laws in an unprimed environment, much like a primary dormant tumor, and escape dormancy at a time proportional to the time when it was disseminated. That is, everything else being equal, if a secondary tumor became initiated 5 years after the initial tumor started growing, it would appear 5 years after the primary tumor was detected, without any additional factors necessarily affecting the dynamics.

Furthermore, while there exists a close clonal relationship between primary and disseminated tumor, the distribution of clones in the disseminated tumor can be very different [45, 54]. In our models, we have shown that changing parameters of the initial distribution of the population of clones in the simulated tumor is sufficient to replicate any variations in time to the escape phase of a simulated dormant tumor.

Based on these considerations, we hypothesize that the logistic distributed or c-distributed Allee models could be more likely for primary tumor dormancy, since they maintain population heterogeneity at an equilibrium state. Due to the gradual nature of the c-distributed Allee model, it might be more suited to account for slowly growing tumors, such as some cases of prostate cancer [44]. In contrast, we propose that the *m*-distributed Allee model would be more likely for metastatic dormancy, since it accounts for decrease of population size to near-zero in the months and years preceding escape from dormancy, and because the decrease in population heterogeneity that occurs at the equilibrium state is consistent with more gradual selection for clones with highest fitness, with the tumor eventually becoming dominated by single clones.

These considerations are summarized in Table 1.

**Table 1:** Summary of results and possible interpretation of the applicability of various parametrically heterogeneous models to describing the dynamics underlying tumor dormancy

Model	Distr. param.	Escape phase	Expected value of dist. param.	Variance of distr. param. after escape	Interpretation/applicability
Malthusian	*c*	rapid	Increases rapidly during escape phase to maximum possible value	Increases rapidly during escape, then returns to zero	Likely not applicable
Logistic	*c*	rapid	Increases rapidly during escape phase to sub-maximum value (inv. proportional to variance)	Increases rapidly during escape, then remains at a non-zero value	Primary tumor dormancy
Allee *x*_*c*_ ' = *cx*_*c*_(*l* − *N*)(*N* − *m*),	*c*	gradual	Increases rapidly during escape phase to sub-maximum value (inv. proportional to variance)	Increases rapidly during escape, then remains at a non-zero value	Slowly-growing tumor
*x*_*l*_ ' = *cx*_*l*_(*l* − *N*)(*N* − *m*),	*l*	less gradual	Increases rapidly during escape phase to maximum possible value	Increases rapidly during escape, then returns to zero	Likely not applicable
*x*_*m*_ ' = *cx*_*m*_(*l* − *N*)(*N* − *m*),	*m*	rapid	Increases rapidly during escape phase to sub-maximum value (inv. proportionate to variance)	Increases rapidly during escape, then gradually decreases	Metastatic tumor dormancy

### Tumor dormancy as part of a larger process

It is possible that for a class of tumors, a series of mutations would lead to the appearance of a population that would grow according to one of the models, proposed here. From our investigation, a parametrically heterogeneous logistic model appears the most likely to describe dormant tumor behavior prior to reaching the carrying capacity defined by spatial and nutrient limitations. This model can account for a rapid escape phase after a long period of latency, as the population grows according to its own “internal clock”, taking many months and years to reach a clinically relevant size, and it allows maintaining population heterogeneity even after the escape phase, when the tumor has reached its current possible carrying capacity. Behavior consistent with escape from dormancy in this case can happen very rapidly, preceded by a dramatic increase in variance and the expected value of the proliferation parameters.

At this time, the tumor might become large enough to start affecting its microenvironment in various ways, thus potentially increasing its carrying capacity [36], allowing it to reach clinically detectable size, since the diameter of tumors at diagnosis is around 1–10 cm [55].

**Fig. 11:**
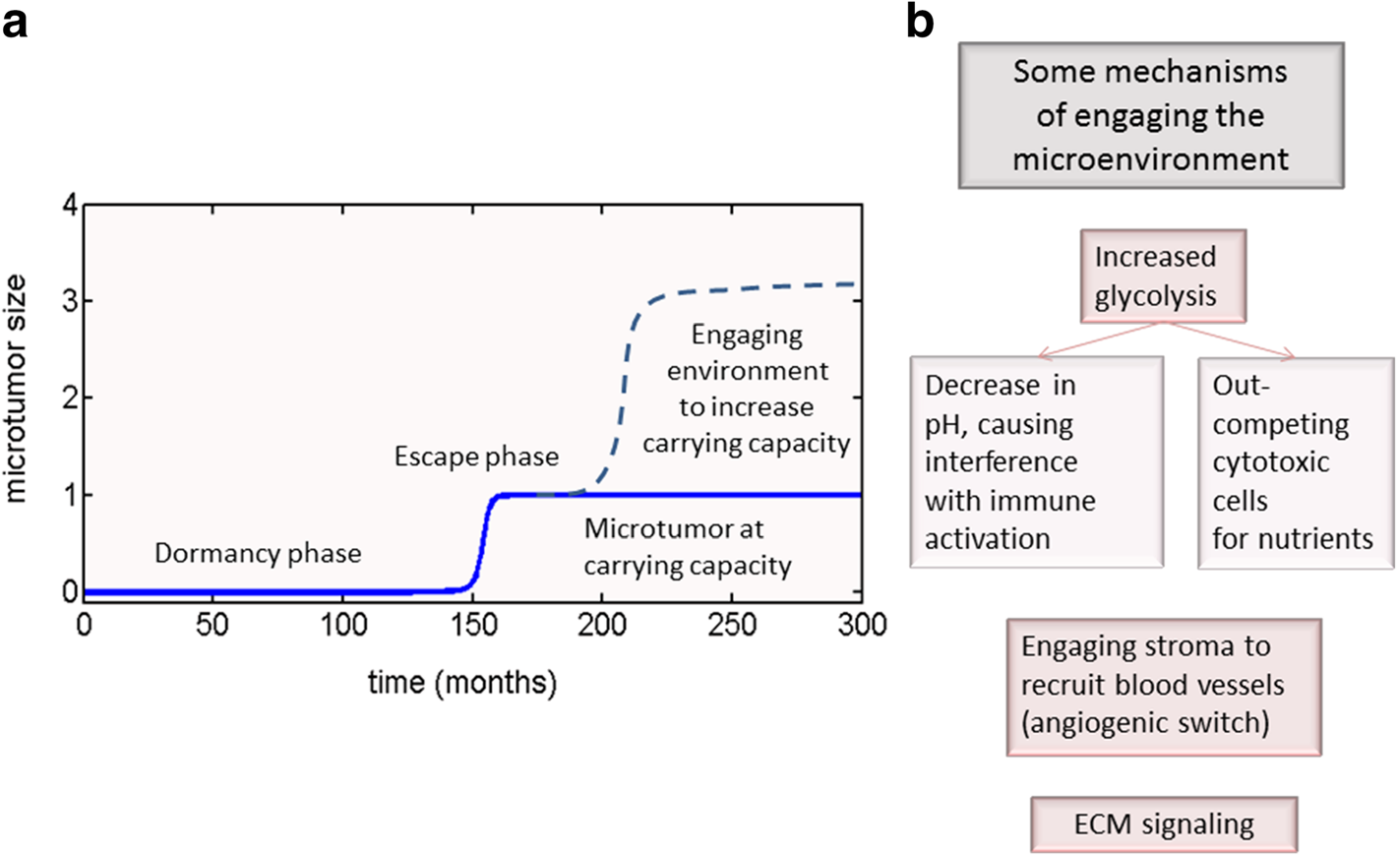
Tumor dormancy as part of a larger process of cancer disease. **a** We hypothesize that at the initial stages of microtumor growth, it may be growing according to parametrically heterogeneous logistic growth law until it reaches an escape phase, which occurs solely due to natural population dynamics. After the escape phase, the tumor may remain indefinitely at its carrying capacity, or it may start engaging it microenvironment to increase its carrying capacity. **b** Some of the mechanisms whereby a tumor can increase its carrying capacity include but are not limited to, increase in glycolytic glucose metabolism, which may lead to interference with immune activation [57], as well as immune cells becoming outcompeted for nutrients [58–60]. The tumor might also engage the stroma to induce angiogenic switch and formation of new blood vessels [22, 24, 56, (Kareva et al.: Normal wound healing and tumor angiogenesis as a game of competitive inhibition of growth factors and inhibitors, under review)], or increase ECM signaling, which has also been implicated in escape from dormancy [15, 27, 66]

One of such mechanisms includes stimulating surrounding stroma to produce angiogenesis regulators that would allow vascularization [16, 22–24, 28, 56, (Kareva et al.: Normal wound healing and tumor angiogenesis as a game of competitive inhibition of growth factors and inhibitors, under review)]. Another mechanism involves exhaustion of oxygen supply and subsequent increase in glycolytic mode of glucose metabolism, which may be followed by local acidosis and down-regulation and starvation of immune system [57–60], allowing for escape from tumor dormancy. Whatever the mechanism that the growing tumor might use to increase its carrying capacity, it engages its environment to foster its own growth, making cancer the systemic disease that it is [57]. The key consideration here is that after the escape phase, the rules that govern tumor dynamics may change to include various aspects of the environment, such as nutrients and predators (immune system), taking the evolution of the system in another direction (see Fig. 11).

Up to the escape phase, it is possible that the initial cluster of mutated cells might simply be growing according to its own laws, and there is nothing that can be done to either induce or prevent escape from dormancy at this stage. However, once the tumor begins growing and engaging it environment, then therapeutic interventions, which take into account the complex nature of both cell populations and of their interactions with their environment [61–64], are most likely to be effective.

More broadly, the proposed investigation highlights the likely possibility that different tumors in different tissues may be described by different growth laws, which may be reflecting both the nature of the underlying tumor and constraints imposed by the environment (i.e., variability in growth rates, death rates, sensitivities to various components of the microenvironment, such as hormone availability, metabolic heterogeneity, etc.). This question can be investigated by fitting different growth functions to tumor growth data and evaluating best fit. A preliminary investigation was recently conducted by Benzekry et al. [65], where the authors investigated goodness of fit of different growth functions to different tumor types. It would be interesting and informative to expand their investigation to include growth functions that account for tumor population heterogeneity, as well as explore what tumor types and what environmental constraints would correspond to which function. If this hypothesis proves to be true, it would be important to use appropriate growth functions in theoretical investigations. Different intervention approaches may become necessary when providing theoretical underpinnings to guiding therapeutic strategies.

## Reviewers’ comments

### Reviewer’s report 1

Heiko Enderling, Moffitt Cancer Center

## Reviewers’ comments

The hypothesis that different growth laws underlie primary and metastatic dormancy is interesting, and mathematical modeling to investigate this applicable. In its current form, the manuscript is difficult to follow and needs more motivation and discussion.

The manuscript discusses three different mathematical models to investigate the plausibility of heterogeneity driving escape from tumor dormancy; Malthusian, Logistic, and Allee growth models. Based on different heterogeneity profiles, tumors escape from dormancy at different time points and with different resulting variations in heterogeneity, from which conclusions are drawn about growth laws underlying either primary or metastatic dormancy. Fairly detailed supplementary data are presented; however, without mathematical training those are difficult to understand.

Without the supplementary data, the manuscript is difficult to follow.

Author’s response: *The supplementary data was incorporated into the main text to facilitate understanding of the proposed hypothesis.*

## Reviewers’ comments

In the main manuscript, parameter c is introduced, but all figures have parameter mu.

Author’s response: *Parameter c is the intrinsic growth rate of the population, described by the proposed growth laws (exponential, logistic, Allee). Parameter mu is the parameter of the initial distribution, in this case truncated exponential. This clarification was added to the text when either of the parameters is first introduced.*

## Reviewers’ comments

The authors argue that it is likely that different intrinsic mechanisms regulate dormancy. Why is this more likely than seed-and-soil?

Author’s response: *Here, I was not suggesting that the proposed framework is more or less likely than the seed-and-soil hypothesis. Rather, I propose an additional, perhaps complementary mechanism that may provide further insights into tumor dynamics during the dormancy phase. One may argue, however, that the proposed model is simpler because it relies solely on intrinsic growth properties of the population and not on specifics of the environment, which may not yet be at play at this stage of avascular tumor growth, and thus requires fewer assumptions to explain the phenomenon of tumor dormancy.*

## Reviewers’ comments

While different mechanisms may very well be at play in primary and metastatic dormant tumors, it is unclear how one can conclude on the likelihood of one growth law over the other from the presented study. It is unclear why different models are applicable.

Author’s response: *A discussion on which of the proposed growth laws may be more or less applicable has been added to the discussion section, and summarized in Table **1*.* For these models, the rationale relies on looking not only at the dynamics of tumor population size but also its composition, in this case accounted for by tracking changes over time of the mean value of the distributed parameter and of its variance.*

*A more thorough investigation, which we are currently developing, would involve fitting data curves found in the literature to various growth functions of heterogeneous population growth and evaluating goodness of fit. A brief discussion of this approach has been added to the end of the manuscript.*

## Reviewers’ comments

The presented hypothesis is that intrinsic growth laws constitute possible mechanisms for escape from dormancy. This hypothesis is insufficiently motivated, and not sure if satisfactorily answered. What is the reason for different proliferation rates in a cancer cell population? Are those rates intrinsic properties or environmental modulation?

Author’s response: *We know from numerous investigations and published accounts that tumors are heterogeneous with respect to various characteristics, presumably resulting from genomic instability. Within the context of the methods investigated here, the characteristics that are being investigated (such as growth rates) are intrinsic and have to be present in the population from the beginning of the simulation. This is a drawback of this method, which has not yet been addressed. That being said, one can argue that certain values of the parameters are present in the initial distribution at such low frequency that their appearance may qualitatively imitate new mutations.*

*The clarification that the values of parameters are intrinsic has been added to introduction of the models.*

## Reviewers’ comments

What range do the proliferation rates span?

Author’s response: *Proliferation rates (or values of any of the other parameters) are given when the initial distribution is defined. According to the principle of maximum entropy (MaxEnt), if the mean value of the random variable is the only quantity that can be estimated from observations or other data, then the most likely distribution of the variable is exponential with the estimated mean. Since no value of a biological characteristic, such as a growth rate, can be infinite, then we should choose the truncated exponential distribution in this interval as the initial distribution. Any other distribution can of course be used, if there exist data or theoretical considerations underlying its choice. This paragraph is highlighted to the main text.*

## Reviewers’ comments

If proliferation rate equals cell fitness, what do cells compete for?

Author’s response: *The answer to this question depends on the model. In the case of exponential growth, cells are unrestrained by resource limitations. In logistic and Allee models, there is a carrying capacity, which represents environmental limitations that would limit population size (whatever they may be, in these particular cases there is no specifications). Since total population size is limited, cells compete for presence in the final population.*

## Reviewers’ comments

Is a spatially averaged model applicable to simulate fitness during avascular dormancy?

Author’s response: *The model considers tumor dynamics at the time when the size of the avascular tumor is still extremely small, in the beginning of its development, so spatial heterogeneity is unlikely to be influencing the dynamics at this stage.*

## Reviewers’ comments

How does heterogeneity impact escape from dormancy?

Author’s response: *As can be seen in the simulations (see, for instance, Figs.* *2*, *3* or *8*), *the initial composition of the population, as determined by parameter mu, correlates to time to escape: larger value of mu (and thus initial distribution skewed more towards smaller values of c) corresponds to longer time to the escape phase. Parameter mu has no physical meaning and is phenomenological. If the data are available, the initial distribution should be estimated from it, with the corresponding distribution parameter.*

## Reviewers’ comments

Is the escape a consequence of heterogeneity, or the reason for heterogeneity?

Author’s response: *In these models, escape is the consequence of heterogeneity. As the population composition changes, clones with higher growth rates become predominant, eventually reaching a critical mass, where all the cells are dividing very rapidly, leading to rapid population growth. This effect is shown particularly in Fig.* *1*.

## Reviewers’ comments

The presented study is without a doubt timely and interesting. I feel, however, that a stronger biological motivation, and more detailed background, materials and methods, explanation and discussion of the results are needed for the study to find the large audience and impact it deserves.

Author’s response: *The text has been significantly expanded compared to its original version, to include more thorough explanation of the methods, a more thorough background, and an expanded explanation of the results and ideas. It is my hope that in its new form, it will achieve these goals.*

### Reviewer’s report 2

Marek Kimmel, Rice University

## Reviewer comments

The paper is based on the interesting idea that delay in metastatic progression may be caused by structured population growth. The paper requires major rewriting before it is suitable for publication. In particular, the supplement should be partly incorporated into the main body (see further on), to make the paper understandable. More detailed medical examples will help.

Major issues - The paper has an unusual construction. Most of the expository material, which clarifies what the author is trying to say, is relegated to a Supplement. This concerns the definition of the parametrically heterogeneous growth laws as well references to the Reduction and Maximum Entropy principles. The relevant part of the Supplement needs to be incorporated in the “Modeling background” or similar section in the main body.

Author’s response: *The paper has been substantially rewritten to facilitate its understanding. It was initially written to fit into the Hypothesis format at Biology Direct, which seemed appropriate due to the theoretical nature of the proposed concept. However, in its expanded form I believe it should be much clearer for the readers.*

## Reviewers’ comments

I am not sure how the paper is related to other papers of Dr. Kareva and her co-author with the same last name. If there is an overlap, it should be carefully discussed.

Author’s response: *Dr. Karev has developed the Reduction Theorem and the theoretical framework for its applications. Over the years we have collaborated on proposing applications. In this work, only the basic aspects of this powerful and elegant theory are used, and only several simple examples are investigated. No claim is made on my part to having come up with novel mathematics. Instead, here I propose a new application for these models. Exponential and logistic models have been previously published in Dr. Karev’s work on demography. To my knowledge, application of the Reduction theorem to Allee models has not yet been published but as one can see, the transformations in all of these cases are not complicated. The novelty of this work is in the proposed applications. This clarification has been added briefly in the text.*

## Reviewers’ comments

The author assembled an interesting, if a little dated (only few entries after 2011) literature concerning biological aspect of metastasis and tumor growth dynamics.

It will make sense to provide some relevant biological context (tumor classifiction, type of study and conclusions; maybe just 1–2 sentences), each time such a source is cited.

Author’s response: *In this work, I do not have access to relevant experimental data. The goal of this work is to propose a mechanism that can provide an additional, possibly complementary, explanation to the phenomenon of tumor dormancy and escape from it. Some experiments were conducted by Almog et al.[*23*]*, *Naumov et al.[*28*]*, *Rogers et al.[*29*]*, *but they were done in mouse xenograph models in the time scales that are not comparable to those investigated in this manuscript. In our following work, we will conduct a more thorough investigation of comparing various growth rate functions to tumors of different tissue origin. A brief discussion of this prospect has been added to the end of the manuscript.*

## Reviewers’ comments

Detailed remarks

p. 2. Please state and explain the Reduction theorem. How is the Reduction theorem different from clonal competition in the context of cancer (if it is similar, then perhaps the connection with driver and passenger mutations should be clarified). p. 3. Sentence “Applying the Reduction theorem (35–37) to …” is not sufficiently informative. Please provide details. Similarly, the paragraph concerning the Allee model is not standing on its own, as of now.

Author’s response: *An explanation of the Reduction theorem has been added to the main text, as well as detailed applications to allow readers to easily follow and reproduce the results for all of the examples, including the Allee model. There is no direct connection that I can currently see between the proposed framework and driver-passenger mutations.*

## Reviewers’ comments

p. 3. I am puzzled as to what the “systemic nature of disseminated disease” (bottom line) might really mean. Please provide details.

Author’s response: *The way that the sentence was phrased was unclear, and it is now altered for clarity. Overall, the comment referred to the fact that cancer in its advanced stages becomes a systemic disease. That is, it engages its environment, changing it, causing a cascade of changes throughout the body, including dysregulation of many organ systems (Redig and McAllister, 2013; Kareva, Waxman and Klement 2015; Sohal, Walsh et al., 2014, among other references on this topic).*

## Reviewers’ comments

Supplement:

p. 1. “…parameter distribution technique, also known as the Reduction Theorem. It was well described in (1, 2), and main results have been summarized in (3).” Unfortunately, this seems not a sufficient explanation.

p. 1. “According to the principle of maximum entropy (MaxEnt)…”. Likewise.

Author’s response: *Explanation for both of these points has been added to the text. An explanation for MaxEnt principle has been put into a separate subsection in order to highlight its importance in estimating the initial distribution in the absence of initial data:*

*“According to the principle of maximum entropy (MaxEnt), if the mean value of the random variable is the only quantity that can be estimated from observations or other data, then the most likely distribution of the variable is exponential with the estimated mean. Since no value of a biological characteristic, such as a growth rate, can be infinite, then we should choose the truncated exponential distribution in this interval as the initial distribution. Any other distribution can of course be used, if there exist data or theoretical considerations underlying its choice.”*

## Reviewers’ comments

p. 2. Calling the expression (3) and (4) the expectation and variance of r.v. c, is misleading if it is not explicitly stated that the distribution of c varies with time (superscript t in the LHS only muddles the issue). Also, is it really needed to call M_0[t], the moment-generating function (mgf)? Are any standard properties of mgf used here?

Author’s response: *Using moment generating function is important in order to easily include the corresponding initial distributions into the investigation. The mgf of the initial distribution is used in the main calculations, as well as its first and second derivatives in order to calculate change over time of expected value of the parameter tracked, and change over time of its variance. In these particular examples, only truncated exponential distribution was used but many other ones can be used as long as their mgf is known.*

*Explicit expressions for calculating expected value and variance as they change over time are given in Eqs. (*3*) and (*4*) to hopefully clarify the origin of the expressions, and to highlight that that do change over time.*

## Reviewers’ comments

p. 3. “… we can observe hyperbolic growth” What does “hyperbolic” mean in this context?

Author’s response: *The term “hyperbolic” here indicates that growth curve in the initial phase is a hyperbola. As one can see from Eq. (*5*), total population size grows in such a way that the Malthusian growth parameter is the expected value of c, which keeps increasing over time, eventually reaching its highest possible value. In this case, it is the value predefined by the interval of the initial truncated exponential distribution. In this growth phase, the population growth is faster than exponential, because the value of Et[c] is increasing. When it reaches it maximal value, the growth becomes exponential. However, to avoid confusion, I removed the word hyperbolic.*
